# Impact of intraoperative fluid management on postoperative complications in patients undergoing minimally invasive esophagectomy for esophageal cancer: a retrospective single-center study

**DOI:** 10.1186/s12871-024-02410-2

**Published:** 2024-01-18

**Authors:** Misaki Takahashi, Hiroaki Toyama, Kazuhiro Takahashi, Yu Kaiho, Yutaka Ejima, Masanori Yamauchi

**Affiliations:** 1 Department of Anesthesiology and Perioperative Medicine, Tohoku University Graduate School of Medicine, 1–1 Seiryo–machi, Aoba–ku, Sendai, 980–8574 Japan; 2 Department of Surgical Center and Supply, Sterilization, Tohoku University Hospital, 1–1 Seiryo–machi, Aoba–ku, Sendai, 980–8574 Japan

**Keywords:** Minimally invasive esophagectomy, Intraoperative fluid management, Postoperative complication, Anastomotic leakage, Postoperative pneumonia, Recurrent nerve palsy, Heart failure

## Abstract

**Background:**

Esophagectomy is a high-risk procedure that can involve serious postoperative complications. There has been an increase in the number of minimally invasive esophagectomies (MIEs) being performed. However, the relationship between intraoperative management and postoperative complications in MIE remains unclear.

**Methods:**

After the institutional review board approval, we enrolled 300 patients who underwent MIE at Tohoku University Hospital between April 2016 and March 2021. The relationships among patient characteristics, intraoperative and perioperative factors, and postoperative complications were retrospectively analyzed. The primary outcome was the relationship between intraoperative fluid volume and anastomotic leakage, and the secondary outcomes included the associations between other perioperative factors and postoperative complications.

**Results:**

Among 300 patients, 28 were excluded because of missing data; accordingly, 272 patients were included in the final analysis. The median [interquartile range] operative duration was 599 [545–682] minutes; total intraoperative infusion volume was 3,747 [3,038–4,399] mL; total infusion volume per body weight per hour was 5.48 [4.42–6.73] mL/kg/h; and fluid balance was + 2,648 [2,015–3,263] mL. The postoperative complications included anastomotic leakage in 68 (25%) patients, recurrent nerve palsy in 91 (33%) patients, pneumonia in 62 (23%) patients, cardiac arrhythmia in 13 (5%) patients, acute kidney injury in 5 (2%) patients, and heart failure in 5 (2%) patients. The Cochrane-Armitage trend test indicated significantly increased anastomotic leakage among patients with a relatively high total infusion volume (*P* = 0.0085). Moreover, anastomotic leakage was associated with male sex but not with peak serum lactate levels. Patients with a longer anesthesia duration or recurrent nerve palsy had a significantly higher incidence of postoperative pneumonia than those without. Further, the incidence of postoperative pneumonia was not associated with the operative duration, total infusion volume, or fluid balance. The operative duration and blood loss were related to the total infusion volume. Acute kidney injury was not associated with the total infusion volume or serum lactate levels.

**Conclusions:**

Among patients who underwent MIE, the total infusion volume was positively correlated with the incidence of anastomotic leakage. Further, postoperative pneumonia was associated with recurrent nerve palsy but not total infusion volume or fluid balance.

## Background

Esophagectomy is a high-risk procedure that is associated with serious postoperative complications, including pneumonia, anastomotic leakage, and cardiac arrhythmia. It is a highly invasive surgery for gastrointestinal cancer given its involvement of extensive surgical fields, including the neck, chest, and abdomen. During esophagectomy, vasopressors are known to impair microvascular blood flow at the gastroesophageal anastomosis. Therefore, esophagectomy involves a liberal fluid protocol and a strongly positive fluid balance in order to maintain gastric microcirculatory blood flow [1, 2]. Restrictive fluid management for maintaining zero fluid balance may prevent pulmonary complications caused by interstitial pulmonary edema after esophagectomy. Accordingly, restrictive fluid management is useful for pulmonary recovery following esophagectomy; further, perioperative fluid balance is positively correlated with the incidence of pneumonia and respiratory failure [3–6]. Additionally, interstitial edema can cause anastomotic leakage and pulmonary complications. In rectal cancer surgery, increased water in the body due to fluid overload can cause edema around the anastomosis and induce leakage [7]. In esophageal cancer surgery, intraoperative fluid overload has been shown to increase the incidence of anastomotic leakage [8]. The Enhanced Recovery After Surgery (ERAS) guidelines for esophagectomy recommend focusing on optimal fluid balance while considering all contributory factors; further, they emphasize the importance of avoiding a positive balance that causes > 2 kg/day weight gain. Goal-directed fluid therapy may be indicated for high-risk patients; however, it is not included in the formal ERAS program. Although these guidelines recommend balanced crystalloids for fluid replacement, they do not specify the proper infusion volume [9].

There has been a recent increase in the number of minimally invasive esophagectomies (MIEs) that adopt thoracoscopic and laparoscopic techniques. MIE is considered less invasive than open thoracotomies; further, it is associated with less blood loss, fewer postoperative complications, a lower incidence of pneumonia within 2 postoperative weeks, lower in-hospital mortality, and significantly lower postoperative serum C-reactive protein levels [10–13]. Although MIE and restrictive fluid management are expected to involve a lower perioperative infusion volume than open thoracotomy, most previous reports only considered open thoracotomy or a combination of MIE with open thoracotomy [8, 14, 15]. Compared with open thoracotomy and/or open abdominal surgery, MIE is considered to involve a lower intraoperative infusion volume. However, the relationship between perioperative infusion volume and postoperative complications in MIE remains unclear.

## Methods

### Study design

This retrospective study was approved by the Ethics Committee of Tohoku University Graduate School of Medicine on December 21, 2021 (#2021–1–850). The need for written informed consent was waived by the Ethics Committee of Tohoku University Graduate School of Medicine due to retrospective nature of the study. Preoperative, intraoperative, and postoperative data were collected from the medical records of patients who underwent MIE for esophageal cancer between April 2016 and March 2021 at Tohoku University Hospital. We excluded patients who underwent procedures other than thoracoscopic esophagectomy, laparoscopic abdominal procedures, or reconstruction using a gastric conduit via the posterior mediastinal route with cervical anastomosis, as well as patients with missing data.

### Perioperative management

All patients were discontinued solid food intake and oral fluid intake 6 and 3 h before admission to the operation room, respectively. All patients underwent MIE under general anesthesia, with or without epidural anesthesia. After induction of general anesthesia, an electromyography endotracheal tube was orotracheally inserted using a video laryngoscope. In addition to the conventional anesthesia monitoring, invasive arterial blood pressure was monitored. Additionally, pulse pressure variation, perfusion index of pulse oximetry, and urine volume were monitored as a fluid management guide. The depth of neuromuscular blockade was maintained at a moderate level (train-of-four count ≥ 1) by adjusting the rocuronium dosage according to acceleromyography or electromyography in order to enable intraoperative monitoring of recurrent laryngeal nerve function. Esophageal dissection was performed with the patient placed in the prone, semi-prone, or left lateral decubitus position using thoracoscopy with or without an artificial pneumothorax. A right-sided chest drainage tube was placed at the end of the thoracoscopic procedure. Subsequently, the patient was switched to the supine decubitus position, and a left-sided chest drainage tube was placed as required. Subsequently, lymph nodes around the stomach were dissected using hand-assisted laparoscopic surgery, followed by construction of a gastric conduit. Simultaneously, cervical lymph nodes were dissected. Next, the gastric conduit was anastomosed to the cervical esophagus via the posterior mediastinum using cervical gastroesophageal anastomosis techniques; moreover, a jejunostomy tube was placed for enteral feeding. Recurrent nerve function was monitored during the upper thoracic and cervical procedures. The attending anesthesiologist adjusted the anesthetic agents, infusion volume, and vasopressors to maintain a mean blood pressure of ≥ 60 mmHg. Arterial blood gas analysis was performed as required. After the surgery was completed, anesthetic administration was discontinued and rocuronium was reversed by an adequate amount of sugammadex until the train-of-four ≥ 110% by acceleromyography or 100% by electromyography; furthermore, the tracheal tube was extubated after recovery from anesthesia, unless there were exceptional circumstances. Subsequently, the patient was transferred to the intensive care unit. On the day after surgery, recurrent nerve palsy was evaluated through fiberoptic laryngoscopy. A week after surgery, anastomotic leakage was evaluated through upper gastrointestinal contrast study and upper endoscopy and CT scan if necessary.

### Data collection

We collected the following patients’ baseline characteristics from the medical records: age, sex, body weight, body mass index (BMI), American Society of Anesthesiologists Physical Status Classification (ASA), preoperative complications of hypertension/diabetes mellitus/chronic obstructive pulmonary disease (COPD)/chronic kidney disease (CKD), radiation therapy, anti-angiogenic therapy, serum total protein and albumin levels, and lymphocyte count. Additionally, we collected data regarding intraoperative factors, including the operative duration, infusion volume, blood loss, urine output, epidural anesthesia, and use of vasopressors. Moreover, we extracted information regarding perioperative factors, including serum lactate levels and body weight, as well as the incidence of postoperative complications, including anastomotic leakage, postoperative pneumonia, acute kidney injury (AKI), recurrent nerve palsy, cardiac arrhythmia, and heart failure. Intraoperative fluid balance was calculated as follows: [infusion volume] + [transfusion volume] – [urine output] – [blood loss]. Postoperative pneumonia was defined as new onset of pneumonia within postoperative 1 month. AKI was defined based on serum creatinine levels following the AKI guideline of Kidney Disease Improving Global Outcomes [16]. Cardiac arrhythmia was defined as the incidence of cardiac arrhythmia requiring treatment intervention or close follow up with electrocardiogram monitoring within postoperative 1 week. Heart failure was defined as new diagnosis of heart failure (International Classification of Diseases, 10th revision [ICD-10] code I50) requiring treatment intervention within postoperative 1 week.

The primary outcome of this study was the relationship between intraoperative fluid volume and anastomotic leakage, and the secondary outcomes included the associations between other perioperative factors and postoperative complications in patients undergoing MIE for esophageal cancer.

### Statistical analysis

Continuous variables are expressed as median [interquartile range] or mean (standard deviation) and were analyzed using Wilcoxon’s rank sum test. Categorical variables are expressed as n (%) and were analyzed using Pearson’s chi-square test. Statistical significance was set at *P *< 0.05. A multivariate analysis was performed using a logistic regression model to evaluate the association between the pre- or intraoperative variables and anastomotic leakage, with the following variables being included: age; sex; body weight; BMI; preoperative serum total albumin and lymphocyte count; preoperative complications of hypertension, diabetes mellitus, chronic obstructive pulmonary disease and/or chronic kidney disease; preoperative radiation therapy; anesthesia and operative duration; blood loss; total infusion volume; fluid balance; and intraoperative total dosage of vasopressors, such as phenylephrine and noradrenaline. Furthermore, propensity score matching was conducted for anastomotic leakage, while the Cochrane-Armitage trend test was performed to determine the dose–response relationship between anastomotic leakage and the intraoperative fluid volume. Additionally, receiver operating characteristic (ROC) analysis between intraoperative fluid volume and the development of anastomotic leakage was performed to estimate the cutoff value of intraoperative fluid volume for predicting the risk of anastomotic leakage. The cutoff value was determined by the Youden index (J); the maximum value of sensitivity plus specificity minus 1, and a goodness of fit for the model was evaluated using chi-squared test. It was calculated that 218 patients were required to demonstrate a difference in intraoperative fluid volume, when the incidence of anastomotic leakage, alpha, beta, and the standard deviation of the intraoperative fluid volume were set to 19%, 5% (0.05), 20% (0.2), and uncertain, respectively. All statistical analyses were performed using the JMP Pro 15 software (SAS Institute Inc., Cary, NC).

## Results

### Patient characteristics, intraoperative factors, and perioperative factors

**Fig. 1:**
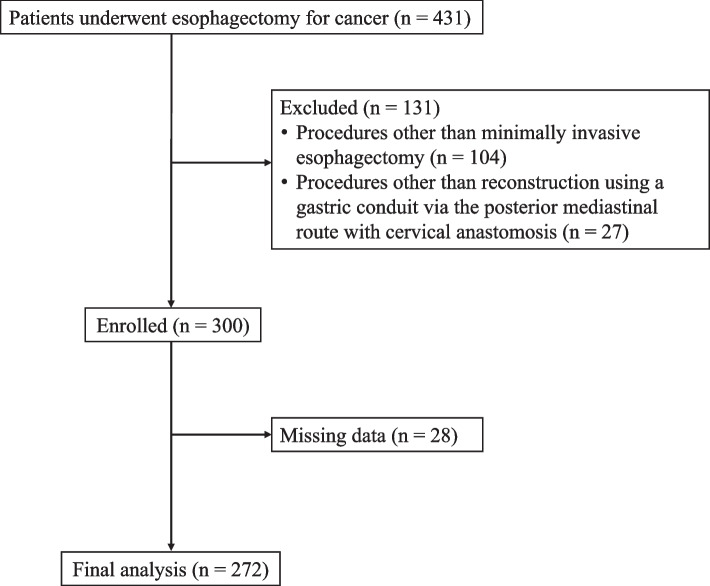
STROBE flow diagram for patient enrollment

Among 300 patients who underwent MIE during the study period, 28 were excluded because of missing data; accordingly, 272 patients were included in the final analysis (Fig. 1). Tables 1 and 2 present the characteristics of the participants and intraoperative management, respectively. The operative duration was 599 [range, 545–682] minutes. The total intraoperative infusion volume, total infusion volume per body weight per hour, and fluid balance were 3,747 [3,038–4,399] mL, 5.48 [4.42–6.73] mL/kg/h, and + 2,648 [2,015–3,263] mL, respectively. Table 3 summarizes the perioperative changes in serum lactate levels and body weights.

**Table 1:** Characteristics of the participants (*n* = 272)

Preoperative factors	Value
Age (years)	68 [62–73]
Sex (male), n (%)	209 (77%)
Body weight (kg)	57.7 [50.1–66.2]
BMI (kg/m^2^)	21.6 [19.6–24.1]
ASA, n (%)	
1	9 (3%)
2	234 (86%)
3	29 (11%)
Hypertension, n (%)	149 (55%)
Diabetes, n (%)	41 (15%)
COPD, n (%)	8 (2.9%)
CKD, n (%)	8 (2.9%)
Radiation therapy, n (%)	25 (9.2%)
Anti-angiogenic therapy, n (%)	0 (0%)
Serum total protein (g/dL)	6.6 [6.2–6.9]
Serum albumin (g/dL)	3.9 [3.6–4.1]
Blood lymphocytes (/µL)	1570 [1175–1955]

Values are presented as median [interquartile range]

*BMI* body mass index, *ASA* American Society of Anesthesiologist physical status, *COPD* chronic obstructive pulmonary disease, *CKD* chronic kidney disease

**Table 2:** Intraoperative management (*n* = 272)

Intraoperative management	Value
Epidural anesthesia, n (%)	266 (98%)
Operative duration (min)	599 [545–682]
Infusion volume (mL)	3747 [3038–4399]
Infusion volume (mL/kg/h)	5.48 [4.42–6.73]
Crystalloid solution (mL)	3159 [2500—3890]
Colloidal solution use, n (%)	199 (73%)
Colloidal solution (mL)	500 [0–1000]
Blood loss (mL)	127 [63–265]
Urine output (mL)	839 [548–1178]
Fluid balance (mL)	2648 [2015–3263]
Fluid balance (mL/h)	225 [166–276]
Use of ephedrine, n (%)	246 (90.4%)
Total amount of ephedrine (mg)	12 [4–20]
Use of phenylephrine, n (%)	267 (98.5%)
Total amount of phenylephrine (mg)	6.0 [3.0–8.9]
Use of noradrenaline, n (%)	25 (9.0%)
Total amount of noradrenaline (μg)	127 (731)

Values are presented as median [interquartile range] or mean (standard deviation)

**Table 3:** Changes in perioperative factors

Perioperative factors		Value
Serum lactate level	Intraoperative peak (mmol/L)	1.9 [1.2–2.8]
	Postoperative day 2	
	Decrease and no change	222 (82%)
	Increase by 0–1 mmol/L, n (%)	47 (17%)
	Increase by > 1 mmol/L, n (%)	3 (1%)
Body weight increase	Immediate after surgery (kg)	+ 1.85 [1.1–2.6]
	Postoperative day 2 (kg)	+ 2.3 [1.5–3.1]

Values are presented as median [interquartile range]

The peak serum lactate level was 1.9 [1.2–2.8] mmol/L; moreover, three (1%) patients showed an increase in the serum lactate value by > 1 mmol/L on the second postoperative day (POD). Compared with the preoperative body weight, the body weight increased by 1.85 [1.1–2.6] kg and 2.3 [1.5–3.1] kg immediately after surgery and on POD 2, respectively.

### Postoperative complications

**Fig. 2:**
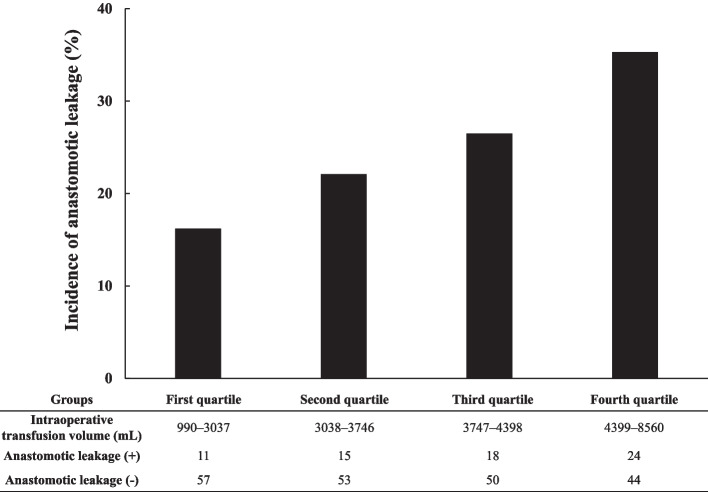
Relationship between the incidence of anastomotic leakage and intraoperative infusion volume. The vertical axis represents the incidence of anastomotic leakage, while the horizontal axis represents the groups sorted according to intraoperative infusion volume. The Cochran–Armitage trend test indicated significantly increased anastomotic leakage in the group with a higher total infusion volume (*P* = 0.0085)

**Fig. 3:**
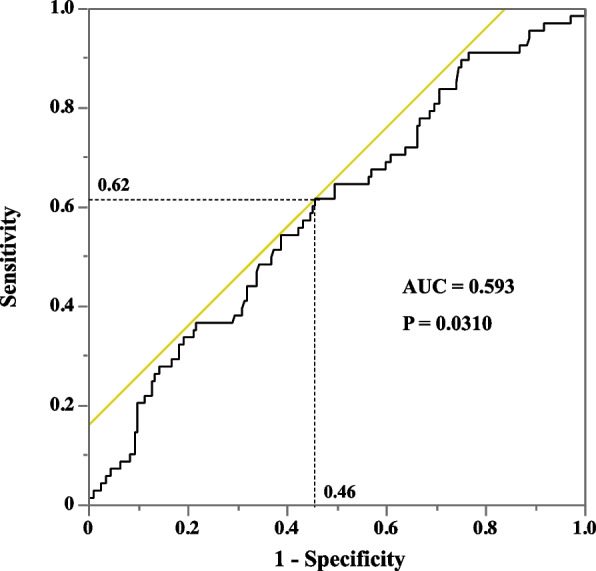
Receiver operating characteristic (ROC) curve of total infusion volume for predicting the risk of anastomotic leakage. ROC analysis was performed to estimate the cutoff value of intraoperative fluid volume for predicting the risk of anastomotic leakage (*n* = 272). The ROC curve had area under the curve (AUC) of 0.59. The cutoff value was estimated to be 3,792 mL, which had a sensitivity of 62% and a specificity of 54% (*P* = 0.0310)

Table 4 shows the incidence of postoperative complications. Anastomotic leakage, recurrent nerve palsy, and pneumonia were observed in 68 (25%), 91 (33%), and 62 (23%) patients, respectively. Table 5 shows the *p*-values of the multivariate logistic regression analysis for the relationships of preoperative and intraoperative factors with postoperative complications. Anastomotic leakage was significantly associated with sex, body weight, and total infusion volume. Table 6 shows the association of the preoperative and intraoperative factors with anastomotic leakage. Anastomotic leakage was significantly more common in male patients (*P* = 0.0005), heavier patients (*P *= 0.0191), and patients with significantly higher total infusion volumes (*P* = 0.0303). However, intraoperative peak lactate levels did not significantly differ between patients with and without anastomotic leakage. Additional propensity score-matching analysis indicated no significant difference in the male sex (*P* = 0.511), the total infusion volume (*P* = 0.7968), and body weight (*P* = 0.7119) between patients with and without anastomotic leakage. However, when the patients were sorted into four quartile groups (each *n* = 68) based on the intraoperative transfusion volume (first quartile, 990–3,037 mL; second quartile, 3,038–3,746 mL; third quartile, 3,747–4,398 mL; and fourth quartile, 4,399–8,560 mL), anastomotic leakage occurred in 11 (16%), 15 (22%), 18 (27%), and 24 (35%) patients in the first, second, third, and fourth quartile groups, respectively. Additionally, the Cochran–Armitage trend test indicated a significant increasing tendency in the incidence of anastomotic leakage in the group with a higher total infusion volume (*P* = 0.0085) (Fig. 2). The ROC analysis showed that the area under the curve (AUC) of the ROC curve was 0.59. The cutoff value of the total infusion volume for predicting the risk of anastomotic leakage was estimated at 3,792 mL, which had a sensitivity of 62% and a specificity of 54% (*P* = 0.0310) (Fig. 3). Moreover, anastomotic leakage was not associated with perioperative serum lactate levels or intraoperative vasopressor use.

**Table 4:** Incidence of postoperative complications

Postoperative complications	n (%)
Anastomotic leakage	68 (25%)
Recurrent nerve palsy	91 (33%)
Pneumonia	62 (23%)
Acute kidney Injury	5 (2%)
Cardiac arrhythmia	13 (5%)
Heart failure	5 (2%)

**Table 5:** The *P*-values of multivariate logistic regression analysis for the relationships of preoperative and intraoperative factors with postoperative complications

Factors	Anastomotic leakage	Postoperative pneumonia	AKI	Cardiac arrhythmia	Heart failure
Preoperative					
Age	0.493	0.115	0.215	0.786	0.0584
Sex	0.0005^*^	0.419	0.866	0.994	0.368
Body weight	0.0191^*^	0.276	0.403	0.425	0.666
BMI	0.520	0.796	0.309	0.611	0.577
Hypertension	0.439	0.569	0.503	0.616	0.0130^*^
Diabetes	0.625	0.792	0.342	0.446	0.756
COPD	0.0974	0.0626	0.694	0.520	0.694
CKD	0.407	0.314	0.694	0.520	0.694
Radiation therapy	0.716	0.880	0.473	0.848	0.399
Serum TP	0.507	0.422	0.0599	0.643	0.610
Serum albumin	0.142	0.0056^*^	0.0168^*^	0.484	0.297
Blood lymphocytes	0.360	0.737	0.126	0.882	0.906
Intraoperative					
Anesthesia duration	0.456	0.0481^*^	0.484	0.984	0.414
Operative duration	0.406	0.0513	0.522	0.977	0.361
Urine output	0.146	0.599	0.0310^*^	0.330	0.0077^*^
Blood loss	0.152	0.111	0.217	0.855	0.715
Infusion volume	0.0303^*^	0.302	0.897	0.161	0.410
Fluid balance	0.247	0.294	0.611	0.0354^*^	0.606
Vasopressors					
Phenylephrine	0.791	0.690	0.359	0.658	0.983
Noradrenaline	0.102	0.996	0.802	0.991	0.726
Peak lactate	0.690	0.857	0.637	0.583	0.0587
Postoperative					
BW change	0.745	0.653	0.877	0.743	0.940
RN palsy	0.207	0.0046^*^	0.754	0.695	0.204

*AKI* acute kidney injury, *BMI* body mass index, *COPD* chronic obstructive pulmonary disease, *CKD* chronic kidney disease, *TP* total protein, *BW* bodyweight, *RN* recurrent nerve

^*^Indicates *P* < 0.05

**Table 6:** Associations of preoperative and intraoperative factors with anastomotic leakage

Factors	Anastomotic leakage ( +) (*n* = 68)	Anastomotic leakage (–) (*n* = 204)	*P* value
Sex (male), n (%)	49 (90.7%)	147 (72.0%)	0.0005^*^
Body weight (kg)	60.9 [51.6–68.1]	56.3 [49.3–65.6]	0.0191^*^
Total infusion volume (mL)	3995 [3239–4782]	3594 [2970–4300]	0.0303^*^
Colloidal solution (mL)	500 [400–1000]	500 [0–1000]	0.0548
Infusion volume (mL/kg/h)	5.6 [4.4–6.9]	5.4 [4.4–6.7]	0.8982
Fluid balance (mL)	2641 [2161–3444]	2651 [1983–3232]	0.2472
Fluid balance (mL/kg)	44.5 [34.6–57.6]	44.8 [33.9–58.8]	0.8423
Lactate (mmol/L)	1.3 [0.9–1.7]	1.3 [0.9–1.9]	0.6899
Total dosage of Ephedrine (mg)	12 [5–20]	12 [40–20]	0.9944
Phenylephrine (mg)	6.2 [3.0–9.4]	5.8 [2.9–8.8]	0.7905
Noradrenaline (μg)	262 (1347)	82 (326)	0.1020

Values are presented as median [interquartile range] or mean (standard deviation)

^*^Indicates *P* < 0.05

Table 5 also shows the secondary outcomes. postoperative pneumonia was associated with an extended anesthesia duration and recurrent nerve palsy. Low preoperative serum albumin levels were significantly associated with postoperative pneumonia and AKI. Postoperative AKI was not associated with the total infusion volume or perioperative serum lactate levels. Patients with postoperative cardiac arrhythmia had a significantly positive fluid balance. Postoperative heart failure was significantly more common in patients with hypertension and low intraoperative urine output. The use of vasopressors was not associated with postoperative complications. Table 7 shows the associations of preoperative and intraoperative factors with total infusion volume. The total infusion volume was associated with the preoperative serum albumin level, lymphocyte count, operative duration, and blood loss. Table 8 summarizes the associations of preoperative and intraoperative factors with postoperative pneumonia. Pneumonia occurred at 4 [2–7.5] postoperative days; further, it was associated with lower preoperative serum albumin levels, a longer anesthesia duration, and recurrent nerve palsy. However, postoperative pneumonia was not associated with the operative duration, total infusion volume, and fluid balance. Table 9 summarizes the associations of preoperative and intraoperative factors with AKI. Postoperative AKI was associated with lower preoperative serum albumin levels but not the total infusion volume. Cardiac tachyarrhythmia requiring medical treatment was observed in 13 (5%) patients. Intraoperative fluid balance was significantly higher in patients with cardiac tachyarrhythmia than in patients without cardiac arrhythmia (3,049 [2,778–3,576] mL vs. 2,521 [2, 3] mL; *P* = 0.0354). Table 10 summarizes the associations of preoperative and intraoperative factors with postoperative heart failure. Five patients were diagnosed with postoperative heart failure. All these patients had hypertension; further, patients with heart failure had significantly lower intraoperative urine output than patients without heart failure. Diabetes mellitus was not associated with postoperative complications.

**Table 7:** Associations of preoperative and intraoperative factors with total infusion volume

Factors	95% confidence interval	*P* value
Age	–0.1533–0.0843	0.5653
Male	–0.0198–0.2158	0.1019
Preoperative albumin	0.0151–0.2487	0.0274^*^
Preoperative lymphocytes	0.0110–0.2449	0.0324^*^
Hypertension	–0.0863–0.1513	0.5879
Diabetes	–0.2247–0.0104	0.0736
Operative time	0.3618–0.5497	< 0.001^*^
Blood loss	0.2770–0.4803	< 0.001^*^

^*^Indicates *P* < 0.05

**Table 8:** Associations of preoperative and intraoperative factors with postoperative pneumonia

Factors	Pneumonia ( +) (*n* = 62)	Pneumonia (–) (*n* = 210)	*P* value
Preoperative albumin (g/dL)	3.8 [3.4–4.0]	3.9 [3.7–4.2]	0.0056^*^
Operative duration (min)	627 [564–693]	634 [559–706]	0.0513
Anesthesia duration (min)	733 [672–795]	695 [637–779]	0.0481^*^
Total infusion volume (mL)	3894 [3110–4692]	3720 [3035–4353]	0.2912
Fluid balance (mL)	2773 [2046–3417]	2619 [2008–3235]	0.3247
Recurrent nerve palsy, n (%)	30 (48%)	61 (29%)	0.0046^*^

Values are presented as median [interquartile range]

^*^Indicates *P* < 0.05

**Table 9:** Associations of preoperative and intraoperative factors with acute kidney injury

Factors	AKI ( +) (*n* = 5)	AKI (–) (*n* = 267)	*P* value
Preoperative albumin (g/dL)	3.6 [2.8–3.8]	3.9 [3.7–4.1]	0.0168^*^
Total infusion volume (mL)	4594 [2157–4900]	3743 [3047–4387]	0.8431
Fluid balance (mL)	3625 [1588–3879]	2643 [2035–3235]	0.5186
Lactate (mmol/L)	1.2 [0.9–1.8]	1.3 [0.9–1.8]	0.9424

Values are presented as median [interquartile range]

*AKI* acute kidney injury

^*^Indicates *P* < 0.05

**Table 10:** Association of preoperative and intraoperative factors with postoperative heart failure

Factors	Heart failure ( +) (*n* = 5)	Heart failure (–) (*n* = 267)	*P* value
Hypertension, n (%)	5 (100%)	118 (44%)	0.0130^*^
Urine output (mL)	370 [260–675]	842 [554–1185]	0.0077^*^

^*^Indicates *P* < 0.05

## Discussion

Esophagectomy is among the most invasive and high-risk gastrointestinal cancer surgeries; further, it is associated with a high rate of serious postoperative complications, including pneumonia, anastomotic leakage, and cardiac arrhythmia. According to the Esophageal Complications Consensus Group, the most common complication after esophagectomy for esophageal cancer is pneumonia (29%), followed by anastomotic leakage (19%) [17], which is consistent with our findings. Kubo et al. assessed the impact of perioperative fluid balance on postoperative complications following MIE for esophageal cancer and reported that acute pneumonia within 7 postoperative days as well as anastomotic leakage were more common in patients with a fluid balance > 3,000 mL on POD 1 but not in patients with a positive fluid balance on the day of surgery [18]. In this previous study, the higher fluid balance group was defined by a fluid balance > 3,000 mL, which is much less than the previously reported fluid balance of 6,900–7,873 mL in patients undergoing right-sided thoracotomy [5, 8]. This indicates that the fluid volume should be strictly controlled on the first POD [18]. Hikasa et al. examined the association between fluid balance and postoperative complications in MIE [19]. They divided the patients into those with a fluid balance > or < 4,311 mL and compared the incidence of postoperative complications. They found that the incidence of postoperative complications, including arrhythmia, deep venous thrombosis, other thromboses, and pneumonia, was significantly higher in patients with a fluid balance > 4,311 mL than in those with a fluid balance < 4,311 mL [19].

In our study, the infusion volume was not directly associated with anastomotic leakage, which may be attributed to the lower total intraoperative infusion volume (3,747 mL) and fluid balance (2,648 mL) compared with previously reported values [5, 8, 19]. Additionally, the Cochrane-Armitage trend test showed increased anastomotic leakage in the group with a higher total infusion volume. And the ROC analysis showed that the total infusion volume of 3,792 mL was the cutoff value for predicting the risk of anastomotic leakage, while the value had low sensitivity and specificity. Excess fluid administration should be avoided; further, it might be better to reduce the total infusion volume below 3,800 mL, although situations which increase total infusion volume are believed to cause anastomotic leakage. In our study, prolonged operative time and increased blood loss were actually associated with an increased total infusion volume; therefore, they may induce anastomotic leakage. Since anastomotic leakage was not associated with the intraoperative use of vasopressors, vasopressors can be appropriately used to reduce the transfusion volume during prolonged operations without increased blood loss. In our study, the combination use of vasopressors and fluid management guides such as pulse pressure variation, perfusion index of pulse oximetry, and urine volume was thought to reduce the total intraoperative infusion volume. Furthermore, high serum lactate levels were not associated with the transfusion volume or anastomotic leakage. This suggests that maintaining adequate blood pressure and preventing inadequate circulation through vasopressor use, rather than volume overload, could be beneficial in certain situations during MIE. Further studies are warranted to establish an optimal fluid management strategy and clarify the significance of elevated serum lactate levels during the perioperative period in patients undergoing MIE. Previous literatures found the several risk factors such as cardiac morbidity, male sex, age > 70 years, ASA > III, BMI (< 18.5 kg/m^2^ or > 30 kg/m^2^), diabetes mellitus, COPD, hypertension, renal disease, alcohol abuse, radiation therapy, anti-angiogenic therapy, preoperative malnutrition (serum albumin < 3.0 g/dL), and cervical anastomosis for anastomotic leakage [20–23]. In our study, the number of patients with ASA > III, COPD, CKD, radiation therapy, anti-angiogenic therapy, and serum albumin < 3.0 g/dL were very small, which could cause no significant association with the incidence of anastomotic leakage because of low statistical power. Additionally, we could not find the association of age and hypertension with the incidence of anastomotic leakage. It might be because most patients were around the age of 70 and had hypertension. All patients in our study underwent cervical anastomosis, which could increase the incidence of anastomotic leakage compared to that of Esophageal Complications Consensus Group [17]. We could not discuss the influence of alcohol abuse and smoking, because the medical records did not describe them.

Postoperative pneumonia was not associated with intraoperative infusion volume or fluid balance, which, similar to the results regarding anastomotic leakage, may be attributed to the lower total intraoperative infusion volume and fluid balance compared with previously reported values. A short interval between surgery and extubation has been associated with lower morbidity rates [24, 25]. A previous study hypothesized that early extubation might contribute to reduced postoperative pulmonary morbidity since it allows a significantly smaller positive fluid balance, which can be attributed to fewer sedation-related hypotensive episodes [1]. A recent systematic review showed that early extubation (immediately after esophagectomy) did not increase the risk of reintubation, mortality, or complications compared with late extubation (12–36 h after surgery) [26]. In this study, early extubation may have prevented an extra fluid load and contributed to fewer pulmonary complications. However, we found that postoperative pneumonia was associated with preoperative serum albumin levels and recurrent nerve palsy. Further, hypoalbuminemia was associated with postoperative pneumonia, which is consistent with a previous report [27]. In patients undergoing MIE, pneumonia has been associated with postoperative recurrent nerve palsy [28]; furthermore, intraoperative nerve palsy monitoring may reduce the risk of recurrent nerve palsy [29]. Taken together, this result highlights the importance of close intraoperative monitoring of recurrent nerve function.

Compared with a liberal fluid protocol, a restrictive fluid protocol has been associated with a significantly higher incidence of AKI after major abdominal surgeries [30]. However, we observed no association between the infusion volume and incidence of AKI. Further, we observed a significant association between lower preoperative albumin levels and postoperative AKI, which is consistent with previous findings [31]. This suggests that a reduced transfusion volume was not a direct cause of postoperative AKI in patients with MIE. Moreover, our findings indicate the importance of improving preoperative nutritional status to prevent postoperative pneumonia and AKI as well as the importance of reducing the transfusion volume, which could prevent other postoperative complications, including anastomotic leakage and cardiac arrhythmia. Clinical frailty is reported to be a risk factor for adverse outcomes in patients undergoing esophagectomy [32]. Our findings afford collateral evidence that interventions targeting frailty such as improving preoperative nutritional status could improve surgical outcome.

Postoperative heart failure was diagnosed in patients with hypertension and decreased intraoperative urine output, suggesting that this was heart failure with preserved ejection fraction caused by latently impaired left ventricular diastolic performance accompanied by hypertension. Moreover, the increased body water was caused by volume overload with reduced urine output induced by low perfusion pressure. Therefore, it is important to perform a perioperative evaluation of cardiac function, especially left ventricular diastolic function, and to identify the cause of reduced urine output in patients undergoing MIE.

Further studies having clear protocol for perioperative hemodynamic and fluid management are warranted to elucidate the relationships of intraoperative infusion volume, fluid balance, and postoperative fluid management with outcomes and biomarkers, such as proinflammatory cytokines, in patients undergoing MIE.

The present study has several limitations. First, this was a single-center retrospective study with a relatively small sample size; further, anesthesia was managed based on the judgment of the attending anesthesiologist since there are no established protocols for anesthesia management such as fluid management, intraoperative blood pressure and cardiac output management, use of vasopressors, and the measurement timing of serum lactate levels. Second, we did not consider the effect of preoperative chemoradiation therapy or the attending surgeon. The condition of the anastomosis might be influenced by surgical skills, intraoperative events, and the patient’s tissue quality following previous chemoradiation therapy [1].

## Conclusions

Our findings indicate that the increase in total infusion volume was associated with the increase in incidence of anastomotic leakage in patients underwent MIE. Reducing the transfusion volume did not cause postoperative AKI; moreover, maintaining adequate blood pressure and preventing inadequate circulation through the use of vasopressors rather than volume overload could be beneficial. Postoperative pneumonia was associated with recurrent nerve palsy but not total infusion volume or fluid balance. Improving preoperative nutritional status is crucial for preventing postoperative complications. Additionally, preventing recurrent nerve palsy could reduce the development of postoperative pneumonia.

## Data Availability

The datasets used and/or analyzed during: the current study are available from the corresponding author on reasonable request.

## Abbreviations

AKI: Acute kidney injury
ASA: American Society of Anesthesiologists Physical Status Classification
AUC: Area under the curve
BMI: Body mass index
CKD: Chronic kidney disease
COPD: Chronic obstructive pulmonary disease
ERAS: Enhanced Recovery After Surgery
MIE: Minimally invasive esophagectomy
POD: Postoperative day
ROC: Receiver operating characteristic
